# The Identification of Key Genes and Pathways in Glioma by Bioinformatics Analysis

**DOI:** 10.1155/2017/1278081

**Published:** 2017-12-06

**Authors:** Mingfa Liu, Zhennan Xu, Zepeng Du, Bingli Wu, Tao Jin, Ke Xu, Liyan Xu, Enmin Li, Haixiong Xu

**Affiliations:** ^1^Department of Neurosurgery, Shantou Central Hospital, Affiliated Shantou Hospital of Sun Yat-sen University, Shantou 515041, China; ^2^Department of Pathology, Shantou Central Hospital, Affiliated Shantou Hospital of Sun Yat-sen University, Shantou 515041, China; ^3^Department of Biochemistry and Molecular Biology, Shantou University Medical College, Shantou 515041, China; ^4^Institute of Oncologic Pathology, Shantou University Medical College, Shantou 515041, China

## Abstract

Glioma is the most common malignant tumor in the central nervous system. This study aims to explore the potential mechanism and identify gene signatures of glioma. The glioma gene expression profile GSE4290 was analyzed for differentially expressed genes (DEGs). Gene ontology (GO) and Kyoto Encyclopedia of Genes and Genomes (KEGG) analyses were applied for the enriched pathways. A protein-protein interaction (PPI) network was constructed to find the hub genes. Survival analysis was conducted to screen and validate critical genes. In this study, 775 downregulated DEGs were identified. GO analysis demonstrated that the DEGs were enriched in cellular protein modification, regulation of cell communication, and regulation of signaling. KEGG analysis indicated that the DEGs were enriched in the MAPK signaling pathway, endocytosis, oxytocin signaling, and calcium signaling. PPI network and module analysis found 12 hub genes, which were enriched in synaptic vesicle cycling rheumatoid arthritis and collecting duct acid secretion. The four key genes CDK17, GNA13, PHF21A, and MTHFD2 were identified in both generation (GSE4412) and validation (GSE4271) dataset, respectively. Regression analysis showed that CDK13, PHF21A, and MTHFD2 were independent predictors. The results suggested that CDK17, GNA13, PHF21A, and MTHFD2 might play important roles and potentially be valuable in the prognosis and treatment of glioma.

## 1. Introduction

Among the various histological subtypes of brain tumor, glioma is the most common malignant tumor in the central nervous system [1]. Established by the World Health Organization (WHO), it can be classified from grade I to grade IV based on histopathological and clinical criteria [2]. During invasive growth, most gliomas extend processes, resulting in a lack of clear borders between tumor and normal brain tissue, making surgical resection of the entire carcinoma difficult. Currently, imageological examination is the most important diagnostic method, as well as the evaluation of the postoperative curative effect. However, imaging is influenced by many factors, such as radiation injury and surgery that result in poor specificity. It is difficult to achieve early diagnosis and treatment of glioma due to a lack of specificity of auxiliary examination indices, so that many patients can lose the chance for radical excision, thereby increasing the risk for poor prognosis. The 5-year overall survival (OS) of patients with glioblastoma is less than 10% [3]. Therefore, the identification of sensitive and specific biological markers that would help identify patients at a higher or lower risk of death from glioma is of vital importance, not only for a better understanding of the molecular and cellular processes involved in tumorigenesis but also for more effective diagnosis, suitable treatment, and improved prognosis.

Gene expression profiling analysis is a useful method with broad clinical application for identifying tumor-related genes in various types of cancer, from molecular diagnosis to pathological classification, from therapeutic evaluation to prognosis prediction, and from drug sensitivity to neoplasm recurrence [4–6]. However, the use of microarrays in clinical practice is limited by the overwhelming number of genes identified by gene profiling, lack of both repeatability and independent validation, and need for complicated statistical analyses [7]. Therefore, in order to put these expression profiles in clinical practice, it is necessary to identify a suitable number of genes and develop a method that can be operated by routine assay. In this study, we downloaded original data from the glioma microarray in the Gene Expression Omnibus (GEO, http://www.ncbi.nlm.nih.gov/geo/), an online public collection database for registration, which is not only for saving microarray data but also for helping the user query and download. We compare gene expression profiles of tumor cells with normal brain cells in order to identify differentially expressed genes (DEGs). Subsequently, the identified DEGs were screened by using Morpheus online software, followed by gene ontology (GO) and pathway enrichment analysis. After analyzing their biological functions and pathways, we further explored the potential biomarkers for diagnosis and prognosis by survival analysis in two independent datasets in order to gain insight on glioma development and progression at the molecular level.

## 2. Materials and Methods

### 2.1. Microarray Data

We downloaded the gene expression profiles in GSE4290, GSE4412, and GSE4271 from the GEO database. GSE4290 has a total of 180 samples, including 157 cases of glioma (26 astrocytomas, 50 oligodendrogliomas, and 81 glioblastomas) and 23 cases of normal brain tissue, based on the GPL570 platform (Affymetrix Human Genome U133 Plus 2.0 Array) by Fine HA et al. Using the GPL96 platform (Affymetrix Human Genome U133A Array), the GES4412 dataset containing 85 cases of glioma was submitted by Nelson SF; and the GES4271 dataset, containing 100 samples that included 77 cases of primary tumor samples and 23 cases of recurrence, was submitted by Phillips HS et al.

### 2.2. Screen Genes of Differential Expression

The analysis was carried out by using GEO2R, an online analysis tool, for the GEO database, based on R language. We applied analysis to classify the sample into two groups that had similar expression patterns in glioma and normal brain tissue. We defined DEGs as differentially expressed with logFC > 2 or logFC < −2, a criteria as described in the references [8, 9]. An adj. *P* value < 0.05 was considered statistically significant. In addition, we used visual hierarchical cluster analysis to show the two groups by Morpheus online analysis software (https://software.broadinstitute.org/morpheus/) after the relative raw data of TXT files was downloaded.

### 2.3. Gene Ontology and KEGG Pathway Analysis of DEGs

With functions including molecular function, biological pathways, and cellular component, gene ontology (GO) analysis annotates genes and gene products [10]. KEGG comprises a set of genome and enzymatic approaches and a biological chemical energy online database [11]. It is a resource for systematic analysis of gene function and related high-level genome functional information. DAVID (https://david.ncifcrf.gov/) can provide systematic and comprehensive biological function annotation information for high-throughput gene expression [12]. Therefore, we applied GO and KEGG pathway analyses to the DEGs by using DAVID online tools at the functional level. A *P* < 0.05 was considered to have significant differences.

### 2.4. Integration of Protein-Protein Interaction (PPI) Network and Module Analysis

The STRING database is an online tool for assessment and integration of protein-protein interactions, including direct (physical), as well as indirect (functional) associations. STRING version 10.0 covers 9,643,763 proteins from 2031 organisms [13]. We drew DEGs using STRING in order to assess the interactional relationships among the DEGs, then used the Cytoscape software to build a PPI network, employed the plug-in Molecular Complex Detection (MCODE) to screen PPI network modules, and established scores > 3 and nodes > 4 in the MCODE module, function, and pathway enrichment analysis. A *P* < 0.05 was considered statistically significant.

### 2.5. Identification of Biomarkers

Based on the information in the individual MCODE modules, the node with the highest score was selected as the hub gene in GSE4290. Every hub gene was also found in two independent datasets (generation dataset GSE4412, primary tumor samples *n* = 85, and validation dataset GSE4271, primary tumor samples *n* = 77) based on the downloaded raw data files, including the information of gene expression value, overall survival time (OS), and survival state. Statistical analyses were performed using SPSS version 20.0 for Windows (IBM, Chicago, IL). We divided expression values into two groups, high expression and low expression, according to X-tile [14]. The Kaplan-Meier method was used to determine the probability of survival and analyzed by the log-rank test. A *P* < 0.05 was considered statistically significant.

## 3. Results

### 3.1. Identification of DEGs

**Figure 1:**
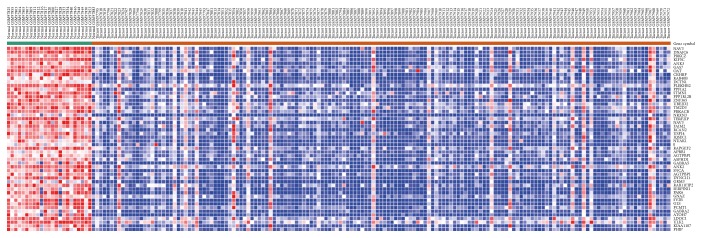
Heat map of the top 50 downregulated genes (red: upregulated; purple: downregulated).

A comparison of 157 cases of glioma samples with 23 cases of normal brain tissue in GSE4290 by using the GEO2R online analysis tool resulted in the identification of the DEGs listed in Figure 1. Based on GEO2R analysis, using an adjusted *P* < 0.05 and log (fold change) (logFC) ≥ 2.0 criteria, there were 775 downregulated genes identified. We further validated the results by using the Morpheus online tool, resulting in a DEG expression heat map, of the top 50 downregulated genes, shown in Figure 1.

### 3.2. Gene Ontology Enrichment Analysis

We used the DAVID online analysis tool to identify statistically significantly enriched GO terms and KEGG pathways after uploading all downregulated genes. GO analysis results showed that downregulated DEGs were significantly enriched in molecular function (MF), including small molecule binding, nucleoside phosphate binding, and carbohydrate derivative binding (Table 1). For biological processes (BP), the downregulated genes were enriched in cellular protein modification, regulation of cell communication, and regulation of signaling (Table 1). In addition, GO cell component (CC) analysis also displayed that the downregulated DEGs were significantly enriched in the cytosol, membrane-bounded vesicles, and nucleoplasm (Table 1).

### 3.3. KEGG Pathway Analysis

The significant enriched pathways of the downregulated DEGs, analyzed by KEGG analysis, are shown in Table 2. The downregulated genes were enriched in the MAPK signaling pathway, endocytosis, oxytocin signaling pathway, calcium signaling pathway, proteoglycans in cancer, purine metabolism, cAMP signaling pathway, and regulation of the actin cytoskeleton.

### 3.4. Module Analysis and Hub Gene Selection in the PPI Network

**Figure 2:**
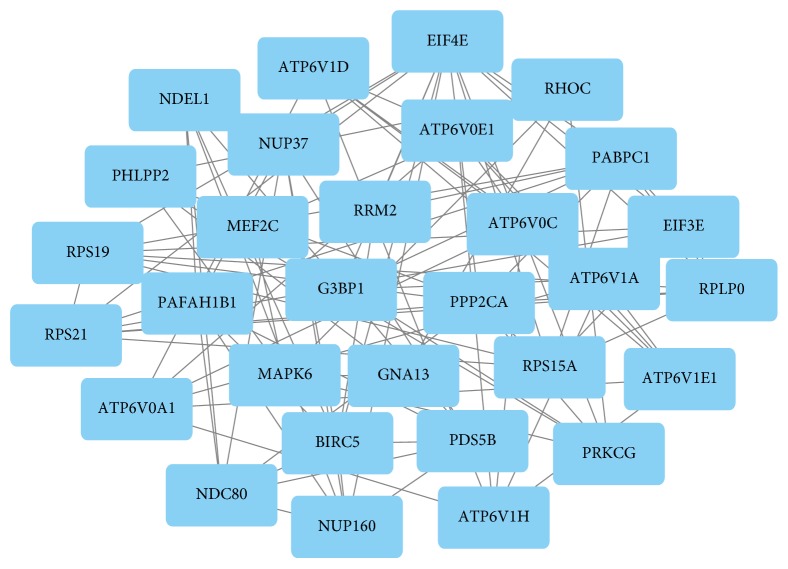
Highest module selected from the PPI network.

Based on the information in the STRING database, the highest module was shown by using the MCODE plug-in, and the functional annotation of the genes involved in the module was analyzed (Figure 2). Enrichment pathway analysis showed that the genes in the module were related to synaptic vesicle cycling, rheumatoid arthritis, and collecting duct acid secretion. Moreover, the 12 hub nodes of the highest score were screened in all modules. The hub genes included PDS5 cohesin associated factor B (PDS5B), chromodomain helicase DNA binding protein 5 (CHD5), cyclin-dependent kinase 17 (CDK17), eukaryotic translation initiation factor 3 subunit E (EIF3E), ATPase H+ transporting V1 subunit H (ATP6V1H), G protein subunit alpha 13 (GNA13), PHD finger protein 21A (PHF21A), methylenetetrahydrofolate dehydrogenase (NADP+ dependent) 2 (MTHFD2), lipoprotein lipase (LPL), adenylosuccinate synthase (ADSS), Wnt family member 10B (WNT10B), and serine and arginine-rich splicing factor 1 (SRSF1). The enriched pathways for genes in the highest module were shown in Table 3.

### 3.5. Identification of Biomarkers

**Figure 3:**
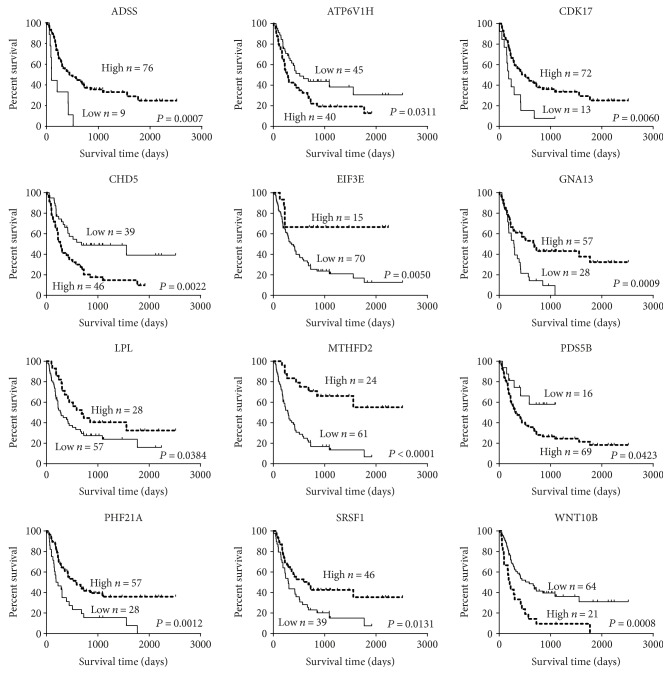
Kaplan-Meier analysis of overall survival for 12 hub genes in the generation dataset of 85 cases.

**Figure 4:**
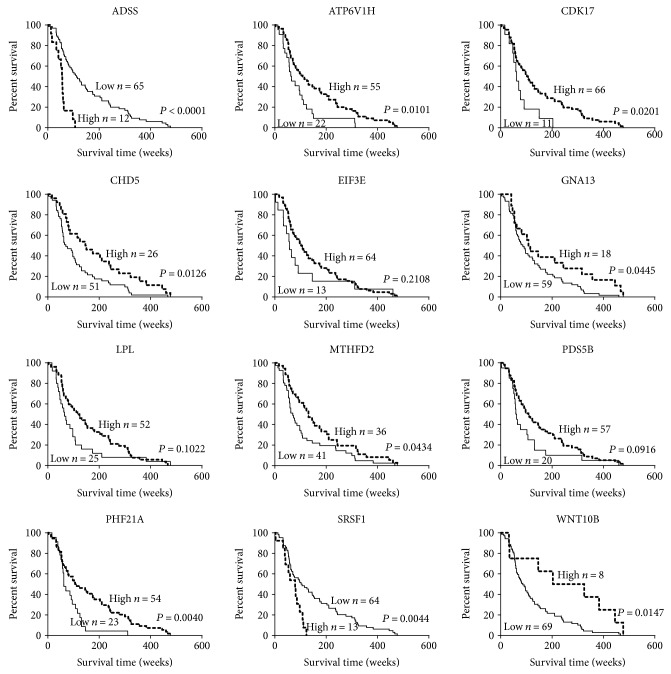
Kaplan-Meier analysis of overall survival for 12 hub genes in the validation dataset of 77 cases.

In order to identify biomarkers, we calculated the survival rate for two groups of 12 hub genes in the generation dataset (GSE4412) and compared the result with the validation dataset (GSE4271) through Kaplan-Meier analysis and log-rank test. According to the analysis, we found that only the downregulation of CDK17, GNA13, PHF21A, and MTHFD2 was closely associated with a decreased overall survival among patients with glioma (Figures 3 and 4). The remaining 8 biomarkers had no statistical significance between gene expression and clinical outcome of glioma or no recoverability in the validation dataset. Furthermore, using the Cox proportional hazards model, a multivariate analysis was performed identifying that expression levels of CDK17, PHF21A, and MTHFD2 were independent prognostic factors (Table 4).

## 4. Discussion

In the present study, we identified DEGs between glioma and normal samples and used a series of bioinformatics analyses to screen key gene and pathways associated with cancer. However, GSE4290 dataset contains only limited number of control samples, 23 out of 180 samples. In order to improve the statistical power of DEG, we defined that the absolute value of the logarithm (base 2) fold change (logFC) greater than 2 and 775 DEGs was obtained. Bioinformatics analysis on DEGs, including GO enrichment, KEGG pathway, PPI network, and survival analyses, found glioma-related genes and pathways that play an important role in cancer initiation and progression.

GO term enrichment analysis demonstrated that 775 downregulated DEGs were significantly enriched in functions involving cellular protein modification, regulation of cell communication, and regulation of signaling. Many studies found that the cellular protein modification, including phosphorylation, ubiquitination, and acetylation, can change the cell biology function, influence disease phenotype, affect glioma cell proliferation, invasion, and apoptosis, and regulate the development of glioma [15–17]. And glioma cells can regulate cell communication, through the information passed to the target cells, interact with the receptor, resulting in specific biological effect such as cell proliferation and cytoskeleton changes, and promote glioma progression and angiogenesis [18]. KEGG pathway analysis indicated that the functions of the downregulated genes were enriched in MAPK signaling, endocytosis, oxytocin signaling, and calcium signaling. Zhang et al. [19] demonstrated that the MAPK signaling pathway induces cell apoptosis in glioma cells and the calcium signaling pathway is involved in quiescence, maintenance, proliferation, and migration in glioma cells [20, 21]. PPI network and module analysis found that the first gene module significantly was enriched in synaptic vesicle cycling. Some results indicate that interference synaptic vesicle cycling could disrupt synaptic function and homeostasis, which would lead to cognitive decline and neurodegeneration in Alzheimer's disease [22]. Therefore, monitoring these signaling pathways may help in the prediction of tumor occurrence and progression.

Since no survival data about GSE4290 could be available, two independent glioma datasets GSE4412 and GSE4271 were applied to detect whether the hub gene could affect the survival time of glioma patients. Survival analysis found that CDK17, GNA13, PHF21A, and MTHFD2 are closely associated with glioma. CDK17 is a member of the cyclin-dependent kinase family. Chaput et al. [23] found that the expression levels of CDK17 are significantly increased in Alzheimer's disease and are associated with the mechanism to promote AD neurodegeneration and may inhibit the pathology development in AD, and Demirkan et al. [24], through a genome-wide association study, found that the CDK17 can be mapped to the glycerophospholipid metabolism pathway. GNA13, one member of the G protein family, is involved in metastasis of tumor cells [25], angiogenesis, and cellular responses to chemokines [26]. In neuronal cells, GNA13 affects neurite outgrowth together with Rho, which is closely related with cell motility and differentiation [27]. Furthermore, GNA13 is coupled to brain-specific angiogenesis inhibitor-1 (BAI1), which is an adhesion-related GPCR, and regulates synaptic function via Rho signaling [28]. PHF21A (also known as BHC80), a plant homeodomain finger-containing protein, can affect the neurofacial and craniofacial development and suppression of the latter and lead to both craniofacial abnormalities and neuronal apoptosis [29]. Moreover, PHF21A specifically binds H3K4me, which is a transcribed genomic locus of regulated posttranslational modification, and implicated the development and maintenance of neural connections [30]. MTHFD2 (methylenetetrahydrofolate dehydrogenase 2) is a mitochondrial enzyme with methylenetetrahydrofolate dehydrogenase and methenyltetrahydrofolate cyclohydrolase activities and has an effect on cancer cell proliferation [31], migration, and invasion [32]. In the expression level of human tumors, MTHFD2 is overexpressed in most cancer types, but exceptions are found in glioma [33], similar to our results. Up to now, the biological functions of CDK17, GNA13, PHF21A, and MTHFD2 in glioma have remained unclear. However, our study shows that the expression level of four key genes are all downregulated in glioma, after comparison with normal brain tissue, and the downregulation is associated with poorer prognosis, as the patients with extended survival time have increased expression of these genes.

At the moment, there are some related bioinformatic research reports of GSE4290 in glioma. Some studies have shown that different enrichment pathway analyses of DEGs can be classified according to their degrees of differential expression during tumor progression in order to explore the deterioration of low into high grade glioma [34]. Some research finds that the DEGs are regulated by transcription factors in glioblastoma [35] and microarray technology has been used to identify the DEGs and their functions in the development of three types of glioma (astrocytoma, glioblastoma, and oligodendroglioma) [36]. Different from these, our study selects the node of the highest score from each module as hub genes in MCODE after comparing nontumor samples with glioma samples. These hub nodes are the key genes of interaction, in the PPI network, that may play important roles in the occurrence and development of glioma. Moreover, hub gene identification is more persuasive, since we validate the association of hub genes and glioma by using survival analysis in two independent datasets to identify four genes that may be cancer biomarkers for glioma. Though not all hub genes associated with the survival of glioma patients, but some hub genes play important roles in immune or inflammation. For example, WNT10B plays an important role in regulating asthmatic airway inflammation through modification of the T cell response [37].

In conclusion, we presumed these key genes identified by a series of bioinformatics analyses on DEGs between tumor samples and normal samples, probably related to the development of glioma. These hub genes could also affect the survival time of glioma patients as validated from two independent datasets. These identified genes and pathways provide a more detailed molecular mechanism for underlying glioma initiation and development. According to the study, downregulation of CDK17, GNA13, PHF21A, and MTHFD2 can be considered as biomarkers or therapeutic targets for glioma. However, further molecular and biological experiments are required to confirm the functions of the key genes in glioma.

## Figures and Tables

**Table 1:** Gene ontology analysis of downregulated genes associated with glioma.

Category	Term/gene function	Count	%	*P* value
GOTERM_MF_FAT	GO:0036094~small molecule binding	119	0.125	2.76*E* − 06
GOTERM_MF_FAT	GO:0000166~nucleotide binding	116	0.121	3.45*E* − 07
GOTERM_MF_FAT	GO:1901265~nucleoside phosphate binding	116	0.121	3.52*E* − 07
GOTERM_MF_FAT	GO:0097367~carbohydrate derivative binding	100	0.105	1.00*E* − 04
GOTERM_MF_FAT	GO:0019899~enzyme binding	94	0.098	2.24*E* − 07
GOTERM_MF_FAT	GO:0017076~purine nucleotide binding	92	0.096	9.44*E* − 06
GOTERM_MF_FAT	GO:0032555~purine ribonucleotide binding	91	0.095	1.26*E* − 05
GOTERM_MF_FAT	GO:0032553~ribonucleotide binding	91	0.095	1.76*E* − 05
GOTERM_BP_FAT	GO:0036211~protein modification process	148	0.155	8.31*E* − 04
GOTERM_BP_FAT	GO:0006464~cellular protein modification process	148	0.155	8.31*E* − 04
GOTERM_BP_FAT	GO:0023051~regulation of signaling	140	0.147	3.36*E* − 07
GOTERM_BP_FAT	GO:0010646~regulation of cell communication	139	0.146	2.25*E* − 07
GOTERM_BP_FAT	GO:0033036~macromolecule localization	138	0.145	5.25*E* − 09
GOTERM_BP_FAT	GO:0006793~phosphorus metabolic process	134	0.140	1.05*E* − 05
GOTERM_BP_FAT	GO:0006796~phosphate-containing compound metabolic process	133	0.139	1.53*E* − 05
GOTERM_BP_FAT	GO:0008104~protein localization	124	0.130	6.40*E* − 09
GOTERM_CC_FAT	GO:0005829~cytosol	163	0.171	4.20*E* − 08
GOTERM_CC_FAT	GO:0031988~membrane-bounded vesicle	152	0.159	2.84*E* − 04
GOTERM_CC_FAT	GO:0005654~nucleoplasm	130	0.136	2.88*E* − 04
GOTERM_CC_FAT	GO:0097458~neuron part	91	0.095	1.38*E* − 11
GOTERM_CC_FAT	GO:0030054~cell junction	75	0.078	1.15*E* − 05
GOTERM_CC_FAT	GO:0043005~neuron projection	66	0.069	1.72*E* − 08
GOTERM_CC_FAT	GO:0005794~Golgi apparatus	66	0.069	0.008029
GOTERM_CC_FAT	GO:0016023~cytoplasmic, membrane-bounded vesicle	64	0.067	7.15*E* − 05

**Table 2:** KEGG pathway analysis of downregulation genes associated with glioma.

Term	Pathway	Gene count	%	*P* value	Genes
hsa04010	MAPK signaling pathway	22	0.023	6.35*E* − 05	MEF2C, BRAF, MAP2K1, NLK, MAP2K4, TP53, PPP3R1, PTPRR, CACNG3, PRKCG, PRKCB, CDC42, RASGRF2, MAPK9, MAPK8IP3, STMN1, PAK1, PRKACB, RAPGEF2, CACNA1C, DUSP7, CACNA1B
hsa04144	Endocytosis	20	0.021	5.98*E* − 04	SH3GL3, PARD3, CLTB, PSD3, PIP5K1C, HLA-E, EPS15, RAB11FIP4, MVB12A, CDC42, AP2A2, RAB31, SH3GLB2, NEDD4, ARPC5L, ARF3, KIAA1033, NEDD4L, IQSEC1, F2R
hsa04921	Oxytocin signaling pathway	15	0.015	5.25*E* − 04	MEF2C, ADCY2, CAMK1G, MAP2K1, PPP1R12B, PPP3R1, CACNG3, PRKCG, CAMKK1, PRKCB, CAMKK2, CAMK2B, GUCY1B3, PRKACB, CACNA1C
hsa04020	Calcium signaling pathway	14	0.014	0.0048	SLC8A1, ADCY2, PTGER3, CCKBR, PPP3R1, PRKCG, PRKCB, ATP2B1,PDE1A, CAMK2B, PRKACB, CACNA1C, CACNA1B, F2R
hsa05205	Proteoglycans in cancer	14	0.014	0.012	CDC42, WNT10B, HIF1A, MAP2K1, BRAF, ANK3, PPP1R12B, TP53, PRKCG, CAMK2B, PAK1, PRKACB, TIMP3, PRKCB
hsa00230	Purine metabolism	13	0.013	0.0109	NME4, ADSS, GDA, ADCY2, POLR1E, RRM2, PDE1A, PRIM2, AK5, GUCY1B3, ENTPD4, HPRT1, GART
hsa04024	cAMP signaling pathway	13	0.013	0.0253	PPARA, ADCY2, PTGER3, BRAF, MAP2K1, ATP2B1, NPY, MAPK9, CAMK2B, PRKACB, PAK1, CACNA1C, F2R
hsa04810	Regulation of actin cytoskeleton	13	0.013	0.0387	GNA13, PAK6, CDC42, ENAH,MAP2K1, BRAF, ARHGEF6, PPP1R12B, ARPC5L, WASF2, PIP5K1C, PAK1, F2R

**Table 3:** The enriched pathways for genes in the highest module.

Pathway	*P* value	FDR	Nodes
Synaptic vesicle cycle	3.36*E* − 08	3.58*E* − 05	ATP6V0C, ATP6V1A, ATP6V0E1, ATP6V1E1, ATP6V1H, ATP6V0A1, ATP6V1D
Rheumatoid arthritis	3.43*E* − 07	3.66*E* − 04	ATP6V0C, ATP6V1A, ATP6V0E1, ATP6V1E1, ATP6V1H, ATP6V0A1, ATP6V1D
Collecting duct acid secretion	2.29*E − *08	2.44*E* − 05	ATP6V0C, ATP6V1A, ATP6V0E1, ATP6V1E1, ATP6V0A1, ATP6V1D

**Table 4:** Cox multivariate analyses of biomarkers associated with OS in the generation and validation datasets.

Dataset	Parameter	Regression coefficient	*P* value	Risk ratio	95% confidence interval
Generation	CDK17	−0.882	0.011	0.414	0.210 ~ 0.815
	MTHFD2	−1.264	0.001	0.283	0.133 ~ 0.598
	PHF21A	−0.671	0.018	0.511	0.293 ~ 0.891
Validation	CDK17	−0.847	0.016	0.429	0.215 ~ 0.856
	MTHFD2	−0.482	0.046	0.617	0.384 ~ 0.992
	PHF21A	−0.620	0.024	0.538	0.314 ~ 0.921
